# Function of the Mitochondrial Transport Protein BcMtp1 in Regulating Vegetative Development, Asexual Reproduction, Stress Response, Fungicide Sensitivity, and Virulence of *Botrytis cinerea*

**DOI:** 10.3390/jof9010025

**Published:** 2022-12-23

**Authors:** Wenyong Shao, Yu Zhang, Changjun Chen, Yujun Xing

**Affiliations:** 1 College of Plant Protection, Nanjing Agricultural University, Nanjing 210095, China; 2 Key Laboratory of Molecular Biology of Crop Pathogens and Insects, Institute of Biotechnology, Zhejiang University, Hangzhou 310058, China; 3 The Key Laboratory for Quality Improvement of Agricultural Products of Zhejiang Province, Department of Plant Pathology, College of Advanced Agricultural Sciences, Zhejiang A&F University, Hangzhou 311300, China; 4 Institute of Food Safety and Nutrition, Jiangsu Academy of Agricultural Sciences, Nanjing 210014, China

**Keywords:** BcMtp1, *Botrytis cinerea*, vegetative development, environmental stress tolerance, virulence

## Abstract

In model fungi, mitochondrial transport proteins (MTPs), also known as “mitochondrial carriers” (MC), are known to facilitate the exchange of biochemical substances across the mitochondrial inner membrane. In this study, we characterized an MTP in *Botrytis cinerea* homologous to the known MTPs in *Saccharomyces cerevisiae* designated BcMtp1. The *BcMtp1* deletion mutant phenotype was strikingly defective in vegetative development, conidiation, and sclerotia production. In addition, *ΔBcMtp1* showed increased sensitivity to osmotic stress, oxidative stress, and cell wall biogenesis inhibitors. In the pathogenicity assay, *ΔBcMtp1* displayed compromised virulence on various host-plant tissues. The *BcMtp1* deletion mutant phenotype was rescued by transforming the wild-type *BcMtp1* variant into the mutant. Together, these data indicate that BcMtp1 is critical for vegetative development, asexual reproduction, stress tolerance, and virulence of *B. cinerea*.

## 1. Introduction

*Botrytis cinerea* causes the gray mold which is responsible for substantial pre- and post-harvest losses in agriculture world-wide. *B. cinerea* is a necrotrophic plant pathogen with more than 1200 host plants [1]. Based on its economic and scientific importance, this pathogen has been rated second among the top ten fungal pathogens [2]. Like most fungi, *B. cinerea* responds to changing environmental cues by alternating between vegetative growth, conidiation, and overwintering stages. However, *B. cinerea* has a diverse arsenal of molecular tools which permit its broad host range and resistance to environmental stress. As a result, *B. cinerea* is able to cause infection in nearly any agricultural context [3]. Therefore, vast challenges are faced across the world in controlling gray mold due to its inherent resilience. The most effective way to improve the control of *B. cinerea* is to improve our understanding of the biochemical mechanisms underlying its growth, reproduction, and virulence [4]. At present, sequencing technology, bioinformatics, and various molecular biotechnology tools have accelerated our understanding of *B. cinerea*, the most important and extensively studied plant-pathogenic fungus [2].

In eukaryotic cells, mitochondria are key organelles involved in energy metabolism, and the regulation of various physiological pathways. These pathways include lipid, sterol, and heme biosynthesis; regulation of programmed cell death (PCD), and many others [5,6,7,8]. In mitochondria, the proton gradient necessary for ATP production is produced by the electron transfer chain (ETC), a respiratory complex which sequentially reduces and oxidizes its components before finally reducing oxygen to form water. This successive redox flux drives protons from the mitochondrial matrix into the intermembrane space. The resulting electrochemical gradient produces the electrical potential which actuates ATP synthase’s (F0-F1 ATP-synthesis complex V) oxidative phosphorylation of ADP into ATP [9,10]. Various mitochondrial transfer proteins exist in the mitochondrial inner membrane, almost all of which are in the mitochondrial carrier family (MCF). MCF proteins catalyze the transport of specific inorganic anions, metabolites, nucleotides and coenzymes across the mitochondrial or peroxisomal membrane [11,12,13,14,15]. These proteins are important regulators of mitochondrial function and produce profound effects on cell physiology. The ADP/ATP carrier (AAC) is one important mitochondrial transfer protein (MTP) involved in the import of ADP into the mitochondrial matrix [16,17]. In *Saccharomyces cerevisiae*, three AAC paralogs are encoded by *AAC1*, *AAC2*, and *AAC3* [18,19]. The expression of *AAC1* can be slightly detected only under aerobic conditions [20], but *AAC3* is expressed only under anaerobic conditions [19]. *AAC2*, by contrast, encodes the major ADP/ATP transporter involved in oxidative phosphorylation, and is required for cell growth on non-fermentation carbon sources, such as ethanol, glycerol, and lactate [21,22].

In the presence of O_2_, the transcription level of AAC1 is very low, and is inhibited during anaerobic growth [23]. AAC1 supports the growth of the AAC2^R96H^ yeast dominant gain-of-function mutant only in aerobic conditions using a multicopy native promoter [20]. However, compared with AAC2, AAC1 is 75% less effective in ADP/ATP transport [18,19]. ADP/ATP transport is most important in yeast under the anaerobic conditions [19]. When the yeast *aac2* deletion mutant is grown under anaerobic conditions, AAC3 function is required for growth [24]. This implies that AAC3 performs the majority of ATP transport during anaerobic growth [19]. In yeast, the AAC2 deletion mutant was compromised in respiratory growth, vegetative growth, and stress tolerance [25].

The MC family in *S. cerevisiae* consists of 35 members, nearly all of which have been functionally characterized. In the *S. cerevisiae* genome, a gene designated *YPR011C* was identified on chromosome 16, which encodes a protein containing motifs characteristic of the mitochondrial carrier family (MCF) [12,26]. YPR011C consists of three repeats of one hundred amino acids. Each repeat contains two transmembrane α-helices punctuated by hydrophilic loop domains [27,28]. YPR011C is known to transport sulfate ions, 3′-phospho-adenosine 5′-phosphosulfate (APS) and almost all proprietary phosphate, by the reverse-exchange mechanism. YPR011C has multiple inhibitors including Bongkrekic acid and other inhibiting factors. In addition, YPR011C transports APS from the inner membrane space to the cytosol under conditions of thermal stress [29].

A BLAST search showed that the *B. cinerea* genome contains a YPR011C homolog, which we designated *BcMtp1*. Based on previous studies of MTPs, we hypothesized that *BcMtp1* might regulate the vegetative development and virulence of *B. cinerea*. We characterized the biological functions of *BcMtp1* in an attempt to verify that hypothesis. However, through a reverse genetics approach, we demonstrate that *BcMtp1* plays a role in the vegetative development, asexual reproduction, stress tolerance, and virulence of *B. cinerea*.

## 2. Materials and methods

### 2.1. Strain, Medium, and Incubation Conditions

The *Botrytis cinerea* strain B05.10 was used as a parent strain for the transformation experiments [30]. All strains in this study were incubated on potato dextrose agar (PDA) (1 L medium contains 200 g potato, 20 g glucose, 26 g agar), minimal medium (MM) (1 L medium contains 10 mM KH_2_PO_4_, 10 mM K_2_HPO_4_, 2.5 mM NaCl, 4 mM (NH4)_2_SO_4_, 0.45 mM CaCl_2_, 2 mM MgSO_4_, 10 mM glucose, 9 μM FeSO_4_, at pH 6.9) or complete medium (CM) (1 L medium contains 2 g peptone, 10 g glucose, 1 g casamino acids,1 g yeast extract, 20 mL nitrate salts, 0.1 mL vitamins trace elements, and pH 6.5) for mycelia growth analysis. Additionally, for the conidiation assay, each strain was incubated on autoclaved potato slices, and PDA.

Mycelial growth inhibition of each strain under different conditions was analyzed on PDA plates supplemented with the following compounds: Congo red, caffeine, NaCl, KCl_,_ paraquat, and H_2_O_2_ at concentrations as shown in the figure caption. A 5mm mycelial plug of each strain cut from the margin of the colony grown on PDA was used to inoculate the 9-mm medium plates. The inhibitory rate of mycelia growth was calculated as [(C − N)/(C − 5)] × 100, where C is the colony diameter of the control and N is the colony diameter of the colony exposed to the experimental treatment. There were three replicates for each treatment. This experiment was repeated three times.

### 2.2. Sequence Analysis of BcMtp1

BcMtp1 (XP_001550923.1) was first identified in the *B. cinerea* genome database (NCBI web site) using BLASTP, with the YPR011C [29] as the query. To analyze the structure of *BcMtp1*, RNA was isolated from mycelia of B05.10 using a commercial RNA isolation kit (Bio-Teke, Beijing, China) and a reverse transcription Kit (TaKaRa, Kusatsu, Japan), used for cDNA synthesis according to the manufacturer’s instructions. The cDNA coding BcMtp1 was amplified from the above synthetic total cDNA with the primer pair P1/P2 (Table S1), and the PCR product was used for sequencing. Moreover, the homology tween BcMtp1 and its homologous protein in various fungi was analyzed via BLASPT, using the amino acid sequences of BcMtp1 and the phylogenetic tree generated by the neighbor-joining method, with Mega 4.1 software on the basis of the deduced their amino acid sequences. Among these fungi, *Saccharomyces cerevisiae* and *Schizosaccharomyces pombe* represent model fungi; *Candida albicans* represent model pathogenic fungi; *Neurospora crassa* represent model filamentous fungi; *Aspergillus nidulans* represent model pathogenic filamentous fungi; *Fusarium graminearum*, *Fusarium oxysporum*, and *Magnaporthe oryzae*. *Sclerotinia sclerotiorum* represent typical plant pathogenic filamentous fungi.

### 2.3. The Deletion and Complementation of BcMtp1

To investigate the function of *BcMtp1* in *B. cinerea*, we constructed a *BcMtp1* deletion mutant. This gene replacement construct was generated according to the previous method with some modifications [31,32]. First, 1328 bp upstream and 1202 bp downstream flanking fragments of *BcMtp1* were amplified from the B05.10 genomic DNA using P3/P4 and P5/P6 primer pairs, respectively (Table S1, Figure S2). Second, a 1764 bp *HPH* fragment containing the trpC promoter (resistant to hygromycin B) was amplified from the pKHT plasmid using the P7/P8 primer pairs according to established plasmid amplification protocols [33,34]. Third, the two *BcMtp1* flanking fragments and the *HPH* cassette were mixed in a 1:1:3 molar ratio and used as the template for double-joint PCR [35] (Figure S2). A 3645 bp DNA fragment was obtained as a result of the double-joint fusion PCR amplification with the P9/P10 primer pair, and was used for the transformation of B05.10 protoplasts.

To generate and transform *B. cinerea* protoplasts, we modified previously established methods [33]. Mycelial plugs cut from the edge of a 3-day-old colony on PDA were put into YEPD liquid medium (3 g/L yeast extract, 20 g/L glucose, and 10 g/L peptone), and then incubated for 18 h with shaking (25 °C, 170 rpm). Fresh mycelia were isolated using an autoclaved gauze and washed three times with sterilized water. A total of 0.25 g of mycelia were incubated in 10 mL 1.5% lysing enzyme solution (0.6 M KCl, 50 mM CaCl_2_) for 1.5 h at 85 rpm and 30 °C. The solution was filtered through mirror paper to remove the mycelial residues. The protoplasts were washed twice using STC solution (0.05 M Tris, 0.8 M sorbitol, 50 mM CaCl_2_, pH 8.0,) and re-suspended in STC-SPTC solution (STC: SPTC = 4:1) (STC with 40% PEG6000). The protoplast transformation was performed according to previous methods [31]. In the transformation, 10^8^ protoplasts in 500 μL of SPT-SPTC solution, 30 μg of replacement construct, and 15 μL spermidine (5 μM) were combined and left on ice for 90 min. One microliter of SPTC was added into the suspension and incubated for 30 min at 25 °C. Next, the protoplast suspension was poured into 200 mL RM medium (0.5 g/L casein hydrolysate, 0.5 g/L yeast extract, 0.7 M sucrose, and 13 g/L agar) at 43 °C. This medium was then poured into 9 mm petri plates and incubated for 16h at 25 °C, in the dark. The RM plates were then covered with SRM medium (0.5 g/L casein hydrolysate, 1 M sucrose, 0.5 g/L yeast extract, and 12 g/L agarose) with 100 mg/L hygromycin B. The plates were incubated for four days at 25 °C, in the dark. The transformants were transferred to the PDA medium with 100 μg/mL hygromycin B for the PCR identification. In the transformant verification assay, PCR amplification of genomic DNA was performed with different primer pairs (Figure S2A, Table S1), and genomic DNA digested with *DraI* and hybridized with a labelled probe for the Southern-blot analysis (Table S1, Figure S2B).

To verify that the phenotypes of *ΔBcMtp1* were due to the deletion of the *BcMtp1* gene, a complemented strain *ΔBcMtp1C* was generated with the full-length *BcMtp1*. The *BcMtp1* complemented plasmid construct pNEO-Mtp1-C was generated with the pNEO plasmid [33]. The full-length *BcMtp1* fragment containing the 659 bp promoter and 887 bp terminator region was amplified from genomic DNA of B05.10 with the P19/P20 primer pair (Table S1), and subsequently cloned into the *BamHI–HidIII* site of pNEO to construct the complemented construct pNEO-Mtp1-C. After the complemented plasmid pNEO-Mtp1-C was verified by sequencing, the construct was transformed into the *BcMtp1* deletion mutant. The transformation procedure was performed as described above. Then, protoplasts suspension was poured into 200 mL RM medium at 43 °C, poured into 9 mm-petri plate, and incubated for 16 h at 25 °C, in the dark. Then, RM plates were covered with SRM medium with 100 μg/mL neomycin. The plates were incubated for four days at 25 °C, in the dark, and the transformants were then transferred to PDA medium with 100 mg/L neomycin. Isolated genomic DNA was digested with *DraI* and hybridized with a labelled probe for the Southern blot identification (Table S1, Figure S2A).

### 2.4. Standard Molecular Methods

Fungi whole-genomic DNA was isolated according to previous methods [31], and was digested with *DraI* using for the Southern blot assay. Plasmid DNA was extracted using a commercial plasmid miniprep purification kit (Omega Bio-tek, Norcross, GA, USA). The Southern blot assay of *BcMtp1* in *B. cinerea* was conducted with a commercial Kit (Roche, Germany), using the 674 bp downstream fragment as the probe (Figure S2). There were three replicates for each treatment. The Southern blot was repeated three times.

### 2.5. qRT-PCR

For total RNA isolation, mycelial plugs of each strain were incubated in YEPD for 2 d (25 °C; 175 rpm), in the dark. Total RNA was isolated from the mycelia of each strain using a commercial kit (Bio-Teke, Beijing, China). The cDNA synthesis was conducted with a commercial reverse transcription kit (TaKaRa, Kusatsu, Japan). The qRT-PCR assay was performed using the ABI 7500 analysis equipment (Applied Biosystems, Waltham, MA, USA) [31,33]. The primer pairs used for qRT-PCR analysis are listed in Table S1. The expression levels of the target gene were normalized to the expression of internal reference gene *actin*, and relative changes in gene expression levels were analyzed with ABI software (Applied Biosystems, Waltham, MA, USA). There were three replicates for each treatment. This experiment was repeated three times.

### 2.6. Fungicide Sensitivity Assay

To analyze the sensitivity of each strain to fungicides, a 5 mm mycelial plug was cut from the edge of fresh colonies of each strain and incubated on PDA plates amended with fluazinam, tebuconazole, fludioxonil, and boscalid. Concentrations of each fungicide are indicated in table caption. After incubation for 3 d at 25 °C, in the dark, the colony diameter was measured, and an EC_50_ value for fungicides against each strain was determined, using DPS software (Reifeng Information Technology, Hangzhou, China) [36]. There were three replicates for each treatment. This experiment was repeated three times.

### 2.7. Glycerol Accumulation Assay

Each strain was used to inoculate YEPD liquid medium. This was incubated for 36 h (25 °C, 175 rpm) in the dark, and either untreated or treated with 0.7 M NaCl for 2 h. After this period, the mycelia of each strain were isolated and ground into powder using liquid nitrogen. The powder (0.2 g) was put into a 2 mL centrifuge tube containing 0.8 mL glycerol isolation solution (Applygen). The tubes were vortex three times for 45 s each and centrifuged at 9000 rpm for 35 min. Then, 0.01 mL of supernatant from this centrifugation was mixed with 0.19 mL detection solution in a new tube. This mixture was incubated at 37 °C for 20 min. The glycerol concentration was analyzed using the microplate reader system at 550 nm (SpectraMax M5) [33,37]. There were three replicates for each treatment. This experiment was repeated three times.

### 2.8. Virulence Assays

To research the role of *BcMtp1* virulence in *B. cinerea*, mycelial plugs, taken from the edge of fresh colonies grown on PDA, were used to inoculate the completely unfolded strawberry and cucumber leaves, as well as apple, grape, and tomato fruit. Before inoculation, host plant tissues were damaged with the autoclaved needle to facilitate the infection of the fungi into host plant tissue. The inoculated plant tissues were incubated at 25 °C with 16 h of daylight and 85% humidity. The disease lesion diameter of each leaf and fruit was measured after the time indicated in the figure caption. There were three replicates for each treatment. The experiment was repeated three times.

### 2.9. Statistical Analysis

All analyses were conducted using the statistical software SAS GLM (SAS Institute, Inc., Cary, NC, USA). The EC_50_ of the strains were estimated by linear regression of the log of the colony diameter versus fungicide concentration. Significance tests adopted least significance difference (LSD)and Duncan methods. There were three replicates for each treatment. Bars denote standard errors from three independent experiments, and values on the bars followed by the same letter are not significantly different at *p* = 0.05.

## 3. Results

### 3.1. Identification of the BcMtp1 Gene

Based on the amino acid similarity to the mitochondrial transport protein (MTP) coded by *YPR011C* in *S. cerevisiae*, BcMtp1 (BC1G_10647) was identified using a BLAST search in the genome data of *B. cinerea* (NCBI web site). The primer pair P1/P2 can amplify both the 1282 bp and 984 bp fragments from genomic DNA and cDNA. The 1282 bp and 984 bp PCR product were sequenced and the sizes and sites of introns of *BcMtp1* identified. The *BcMtp1* encoded a 327 amino acid long protein sequence, and the conserved domain was identified in various fungi, which indicated that the Mtp1 was conserved among fungi in the evolution process (Figure S1).

### 3.2. Deletion of BcMtp1 and Complementation

To investigate the function of *BcMtp1* of *B. cinerea*, the *BcMtp1* deletion mutant was generated via homologous replacement tactics (Figure S2). We obtained three *BcMtp1* deletion mutants in 103 hygromycin-resistant transformants with the described PCR amplification method, using specific primer pairs (Figure S2; Table S1). The 459 bp fragment was amplified in the parent strain B05.10 but not in the *ΔBcMtp1*, using the P11/P12 primer pair. A 475 bp fragment amplified from the *HPH* gene was only obtained in *ΔBcMtp1*, using the primer pair P13/P14. Then, the 2677 bp upstream and 2524 bp downstream flanking regions were amplified in *ΔBcMtp1*, but not in the parent strain B05.10, using P15/P16 and P17/P18 primer pairs (Figure S2), respectively. These results suggest that the replacement construct successfully substituted the *BcMtp1* through homologous recombination. In the Southern blot analysis, we probed using a 674 bp 3′-flanking segment of *BcMtp1*. The *ΔBcMtp1* produced a 5.7 kb band that lacked a 3.9 kb band, which was consistently detected in the parent strain parent and complemented strains. These results verified that *ΔBcMtp1* was generated by a single homologous recombination at the *BcMtp1* site, and there was a single copy of the *BcMtp1* in the complemented strain *ΔBcMtp1C*.

### 3.3. BcMtp1 Is Involved in Vegetative Growth

**Figure 1:**
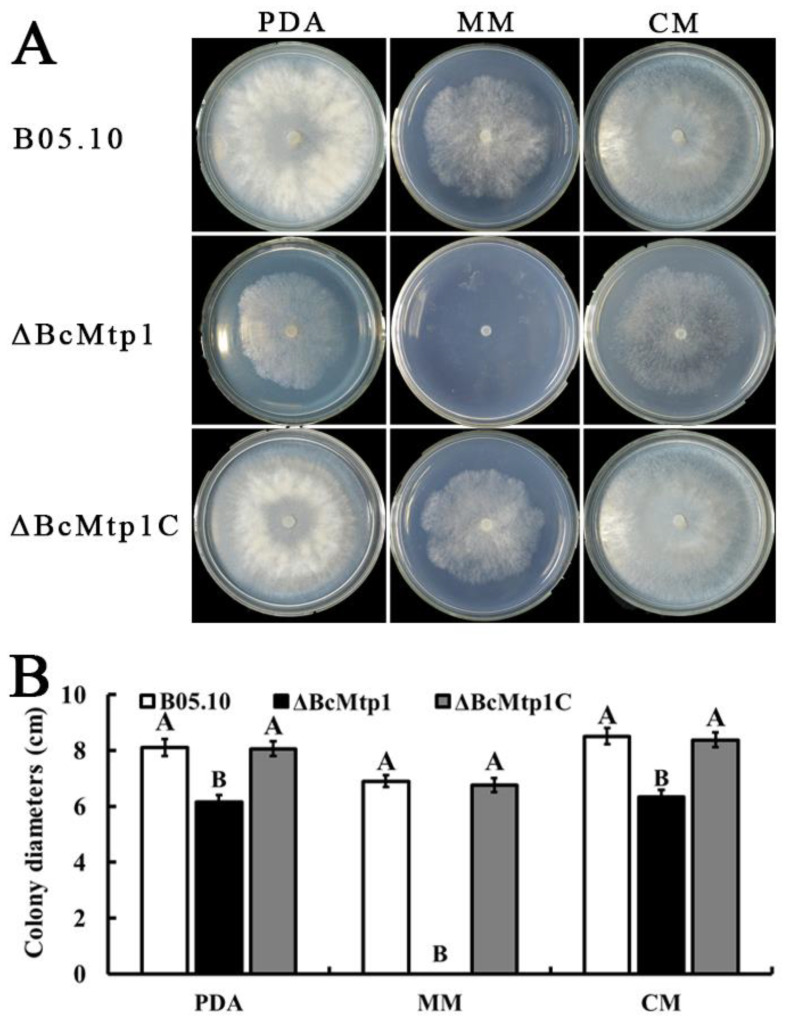
Effect of BcMtp1 deletion on mycelia growth. (**A**) The wild-type strain B05.10, the BcMtp1 deletion mutant *ΔBcMtp1*, and the complemented strain *ΔBcMtp1C* were grown on solid media [potato dextrose agar (PDA), complete medium (CM), and minimal medium (MM)] for 3 d at 25 °C, in the dark. (**B**) The colony diameter of B05.10, *ΔBcMtp1*, and *ΔBcMtp1C* on PDA, CM, and MM for 3 d at 25 °C, in the dark. Bars denote standard errors from three repeated experiments.

Each strain was incubated on PDA, CM, or MM plates for 3 d at 25 °C, in the dark. After this incubation period, the mean colony diameter of each strain was measured. As shown in Figure 1, on the PDA and CM medium, the mycelial growth rate of *ΔBcMtp1* was slower than that of B05.10 and *ΔBcMtp1C*. In addition, B05.10 and *ΔBcMtp1C* grew normally on the MM medium, but *ΔBcMtp1* was unable to grow. These results indicate that BcMtp1 influences vegetative growth processes in *B. cinerea*.

### 3.4. BcMtp1 Is Required for Conidiation and Sclerotia Production

**Figure 2:**
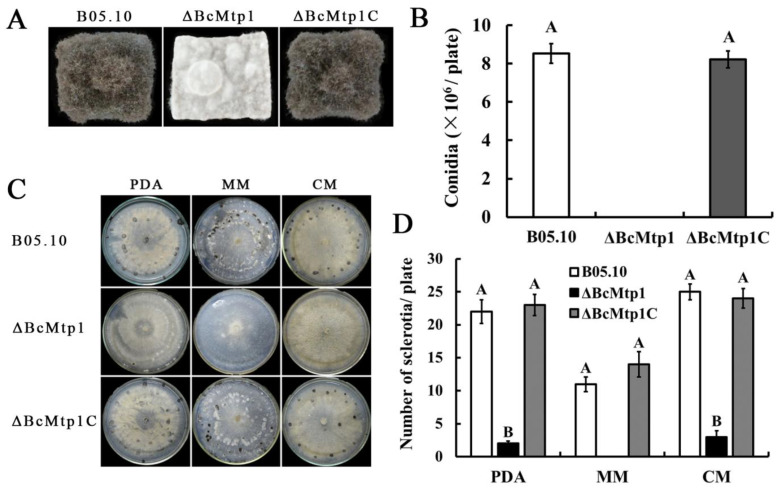
Function of BcMtp1 on conidial and sclerotial formation. (**A**) Comparison of conidiation among B05.10, *ΔBcMtp1*, and *ΔBcMtp1C* after 12 d incubation on sterilized potato plugs at 25 °C in dark. (**B**) The number of conidia produced by each strain on potato dextrose agar (PDA) plates (diameter, 9 cm). Bars denote standard deviation from three experiments. Values on the bars followed by the same letter are not significantly different at *p* = 0.05. (**C**) Comparison of sclerotial formation among B05.10, *ΔBcMtp1*, and *ΔBcMtp1C* after 4 weeks of incubation on PDA, complete medium (CM) and minimal medium (MM) at 25 °C, in darkness. (**D**) The number of sclerotia was measured on PDA, CM, and MM. Bars denote standard deviation from three experiments. Values on the bars followed by the same letter are not significantly different at *p* = 0.05.

To analyze conidiation, each strain was incubated on autoclaved potato plugs for 12 days. B05.10 and *ΔBcMtp1C* produced dense mycelial mats covered with conidia, however, *ΔBcMtp1* only produced sparse mycelia, without any conidia (Figure 2A,B).

We also analyzed the role of *BcMtp1* in sclerotia formation by incubating each strain on PDA, CM, or MM in the dark for four weeks at 16 °C. As expected, B05.10 and the *ΔBcMtp1C* produced extensive sclerotia. By comparison, *ΔBcMtp1* failed to produce any sclerotia (Figure 2C,D). These results demonstrate that *ΔBcMtp1* exhibit compromised conidiation and sclerotia formation in *B. cinerea*.

### 3.5. The Role of BcMtp1 in Stress Response

**Figure 3:**
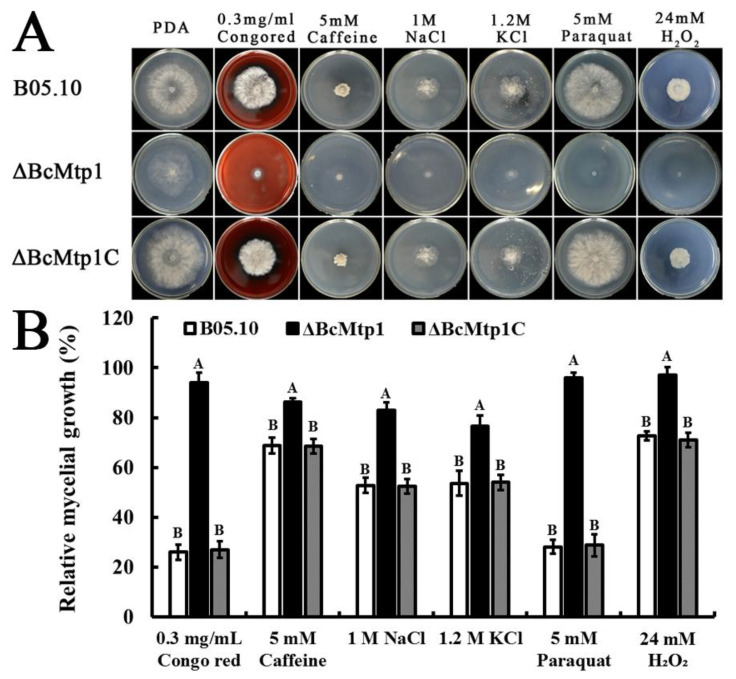
Sensitivity of B05.10, *ΔBcMtp1* and *ΔBcMtp1C* to various stresses. (**A**) Comparisons were performed on PDA medium supplemented with Congo red, caffeine, NaCl, KCl, paraquat, and H_2_O_2_, indicated in the figure. (**B**) Inhibition of mycelial growth was analyzed after each strain was incubated for 3 d on PDA supplemented with different compound, as described in the figure. Bars denote standard deviation from three experiments. Values on the bars followed by the same letter are not significantly different at *p* = 0.05.

After observing vegetative growth defects in *ΔBcMtp1*, we analyzed the sensitivity of *ΔBcMtp1* to various stresses including cell wall biogenesis inhibitors (Congo red and caffeine), oxidative stressors, and osmotic stressors. *ΔBcMtp1* exhibited dramatically increased sensitivity to cell wall-related stress caused by 0.3 g/L or 5 mM caffeine, compared to the parent strain B05.10 and the complemented strain *ΔBcMtp1C* (Figure 3). Additionally, the sensitivity of *ΔBcMtp1* to osmotic stress, generated by 1.0 M NaCl or 1.2 M KCl, and oxidative stress, generated by 5 mM paraquat or 24 mM H_2_O_2_, were significantly increased compared to B05.10 and *ΔBcMtp1C* (Figure 3). The results may indicate that the deletion of BcMtp1 increases the sensitivity of *B. cinerea* to various stresses.

### 3.6. BcMtp1 Is Required for Glycerol Accumulation

**Figure 4:**
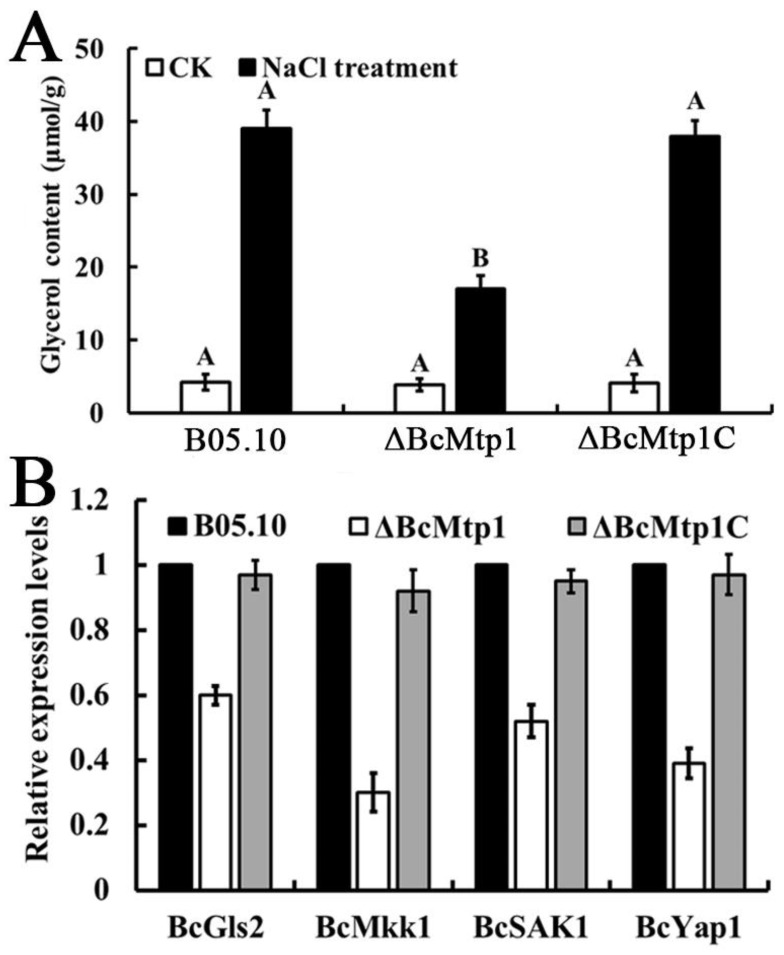
Involvement of BcMtp1 in glycerol accumulation and expression levels of various stress response genes (**A**) Glycerol content in mycelia of B05.10, ΔBcMtp1, and ΔBcMtp1C. The glycerol was extracted from the mycelia of each strain untreated or treated with 0.7 M NaCl (NaCl treatment) for 2 h after growth in YEPD, in the dark. The cultures without treatment were used as controls (CK). Bars denote standard errors from three repeated experiments. Values on the bars followed by the same letter are not significantly different at *p* = 0.05. (**B**) Expression levels of cell-wall integrity relation genes (*BcMkk1* and *BcGls2*) and oxidative stress relation gene (*BcYap1* and *BcSak1*) in each strain. RNA samples were extracted from the mycelia of each strain after being grown in YEPD for 2 d (25 °C, 175 rpm), in the dark. Bars denote standard deviation from three repeated experiments. Values on the bars followed by the same letter are not significantly different at *p* = 0.05.

The glycerol production capacity was analyzed in B05.10, *ΔBcMtp1*, and *ΔBcMtp1C* after treatment with 0.7 M NaCl for 2 h. Low glycerol accumulation was detected in B05.10, *ΔBcMtp1*, and *ΔBcMtp1C* under normal growth conditions. In the experimental trial, NaCl stress clearly stimulated glycerol accumulation in B05.10 and *ΔBcMtp1C*, but no such accumulation was observed in *ΔBcMtp1* (Figure 4A). These results indicated that BcMtp1 may influence glycerol accumulation in *B. cinerea*.

### 3.7. BcMtp1 Is Involved in the Transcriptional Regulation of the Genes Related to Environmental Stress

*BcMtp1* exhibited enhanced sensitivity to cell wall biogenesis inhibitors, indicating that *BcMtp1* might be related to the maintenance of cell wall integrity in *B. cinerea*. We determined the expression level of the *B. cinerea* homologs of *Mkk1 and Gls2*, designated *BcMkk1* and *BcGls2*. *BcMkk1* and *BcGls2* expression were strongly downregulated in the *ΔBcMtp1*, compared to B05.10 and *ΔBcMtp1C*. Moreover, RT-PCR analysis indicated that osmotic stress related *BcSak1* was expressed less in *ΔBcMtp1* compared to B05.10 and *ΔBcMtp1C*, under osmotic stress conditions (Figure 4B). As shown in Figure 4B, the expression level of oxidative stress associated gene *BcYap1* was markedly decreased in *ΔBcMtp1*, relative to B05.10 and *ΔBcMtp1C*. These results demonstrate that BcMtp1 is likely an indirect transcriptional regulator of stress response genes.

### 3.8. Involvement of BcMtp1 in the Sensitivity of B. cinerea to Fungicides

In the fungicide sensitivity assay neither *ΔBcMtp1*, B05.10, nor the complemented strain exhibited a significant difference in their sensitivities to boscalid, tebuconazole, and fludioxonil. Interestingly, the sensitivity of *ΔBcMtp1* to fluazinam was significantly decreased, compared to that of B05.10 and the *ΔBcMtp1C* (Table 1). This might suggest that fluazinam plays some disruptive role on MC protein function.

### 3.9. BcMtp1 Is Required for Virulence of B. cinerea

**Figure 5:**
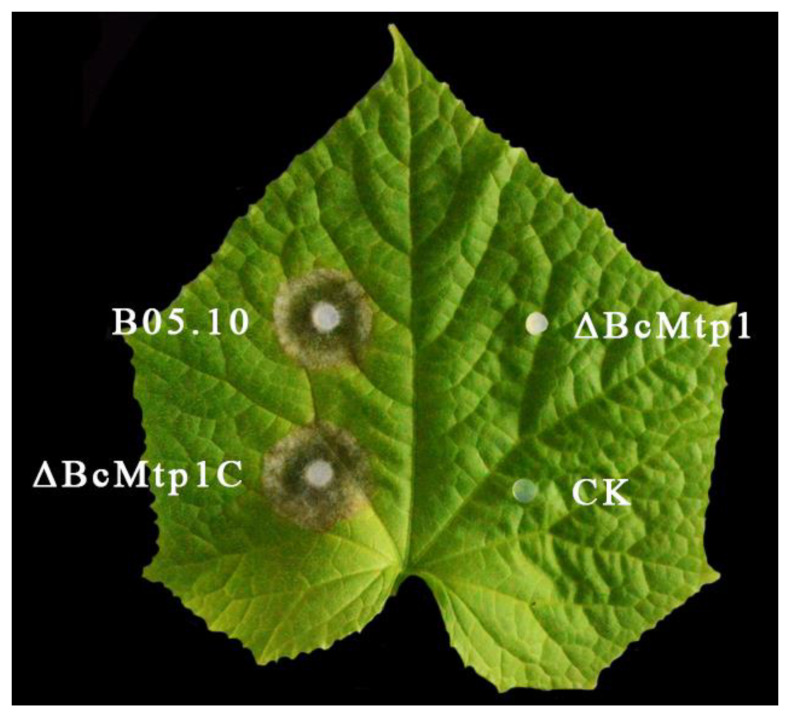
Pathogenicity assays on wounded cucumber leaves following inoculation with the wild-type B05.10 strain, the mutant *ΔBcMtp1*, and the complemented strain *ΔBcMtp1C*. Agar plugs without fungal mycelia were used as negative controls (CK). Disease symptoms were analyzed 60 h post inoculation (h.p.i.).

**Figure 6:**
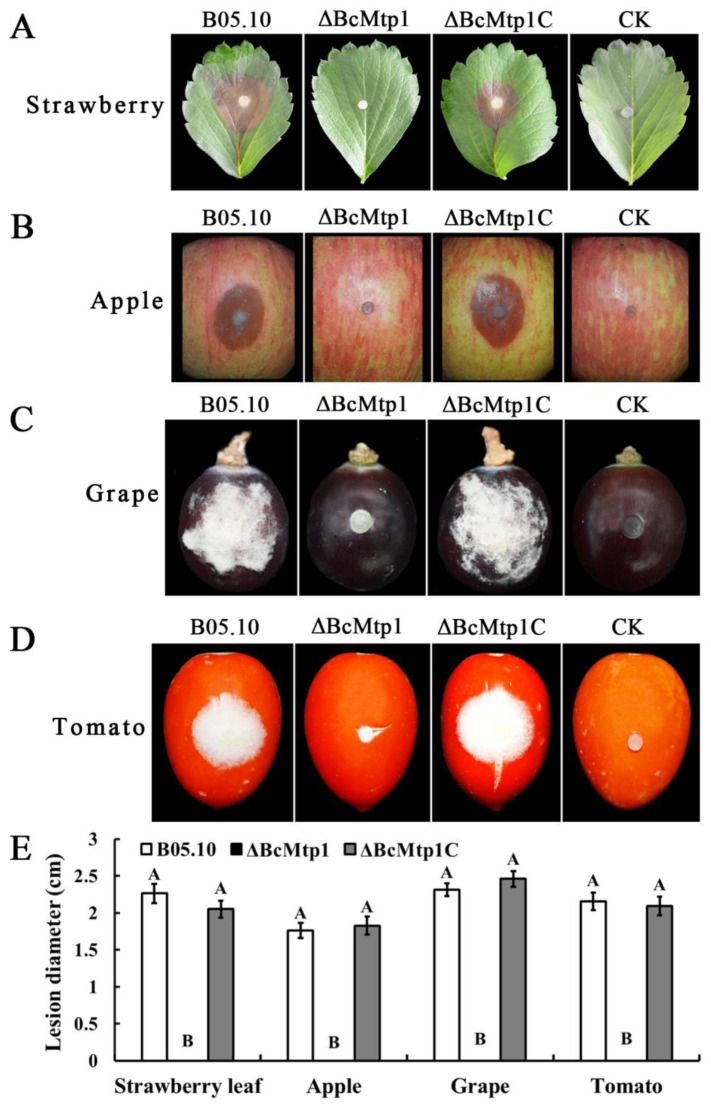
Pathogenicity analysis on various plant tissue, following inoculation with the parental strain B05.10, *ΔBcMtp1*, and *ΔBcMtp1C*. Agar plugs were used as negative controls (CK). (**A**) Disease symptoms on wounded strawberry leaves after 80 h post inoculation (h.p.i.), (**B**) wounded apple fruits 80 (h.p.i), (**C**) wounded grape fruits 72 (h.p.i), (**D**) wounded tomato fruits 72 (h.p.i), and (**E**) Diameter of disease lesion on various plant tissue. Bars denote the standard deviation of four repeated experiments. Values on the bars followed by the same letter are not significantly different at *p* = 0.05.

The effect of *BcMtp1* on virulence of *B. cinerea* was analyzed on various host plant tissues. Since *ΔBcMtp1* was severely compromised in conidiation, we used mycelial plugs instead of spore suspensions to inoculate various host plant tissues in these virulence assays. On wounded leaves of strawberry and cucumber, apple, grape, and tomato, the B05.10 and *ΔBcMtp1C* caused a consistent and expected severity of disease symptoms on host plant tissues. *ΔBcMtp1* was not able to cause any obvious lesions on these host plant tissues compared to the wild-type and complemented strain controls (Figure 5 and Figure 6). These results indicate that BcMtp1 is involved in the virulence of *B. cinerea*.

## 4. Discussion

Mitochondrial carrier proteins are likely a convergent node for various metabolic pathways, as they facilitate the exchange of biochemical compounds across the mitochondrial inner membrane. These data indicate that *BcMtp1* plays a critical role in vegetative development, asexual reproduction, stress tolerance, and virulence. The yeast mitochondrial carrier protein AAC2 was essential for the cell viability, with almost all of laboratory strains showing a lethal phenotype when the AAC2 was disrupted [38,39]. In this study, the *ΔBcMtp1* showed defects in mycelial growth on PDA and CM, and strikingly lost its ability to grow on MM. These results suggest that this mitochondrial carrier protein may be required for vegetative development in several fungi. Asexual reproduction as measured by conidiation also appeared to be compromised.

It is well-established that conidiation is a critical process or secondary infection processes in *B. cinerea* [40]. In addition, the sclerotia produced in dying host tissues is perhaps the most important mechanism underlying the persistent nature of *B. cinerea* [3]. In our study, the results showed that the effects of the deletion of BcMtp1 on the conidia production and the sclerotia development indicate that BcMtp1 is involved in the secondary infection and survival ability of *B. cinerea*, which are essential for the gray mold formation and extension.

In yeast, deletion of *YPR011C* also exhibited decreased stress tolerance (high temperature: 45 °C) [29]. In this study, the results showed that *ΔBcMtp1* exhibited increased sensitivity to various stresses including cell wall biogenesis inhibitors (Congo red and Caffeine), inducers of oxidative and osmotic stress, compared to B05.10 and *ΔBcMtp1C*. This suggests that this mitochondrial carrier protein is involved in mounting an appropriate response to stress. In previous studies, osmotic stress was demonstrated to enhance glycerol accumulation in fungi, primarily through HOG pathway activation [41,42]. The Mkk1 and Gls2 proteins are involved in the maintenance of cell wall integrity in *S. cerevisiae* [43]. The previous study showed that *BcSak1* regulated the sensitivity of *B. cinerea* to osmotic stress [44,45], and Yap1 was a significant regulation factor in the sensitivity of *S. cerevisiae* to oxidative stress [46,47,48]. In this study, we found that the glycerol accumulation and expression of genes related to stress tolerance were significantly lower in *ΔBcMtp1* than in B05.10 and *ΔBcMtp1C*. From this, we inferred that *BcMtp1* might be involved in the sensitivity of *B. cinerea* to environmental stress by regulating the expression of glycerol accumulation and stress response related genes (*BcMkk1*, *BcGls*, *BcSak1*, and *BcYap1*). This conclusion needs to be explored further in subsequent research. The fungicide sensitivity analysis demonstrated the unexpected result that the response of *ΔBcMtp1* to fluzinam was decreased, relative to control. In previous research, the fungicidal effect of fluazinam for fungal pathogens was the disruption of oxidative phosphorylation [49]. Therefore, BcMtp1 may be compromised in overall respiration, leading to the decreased sensitivity to the fluzinam in *ΔBcMtp1*.

In the virulence assay, *ΔBcMtp1* was significantly less virulent on various plant tissues. Whether this is merely a result of the compromised metabolic function observed in this mutant, or if BcMtp1 plays a specific role in virulence remains unclear. Not only was the growth rate of *ΔBcMtp1* slower than B05.10 and *BcMtp1C*, but *ΔBcMtp1* also exhibited increased sensitivity to cell wall biogenesis inhibitors. In previous studies, the cell wall has been demonstrated to be essential for the ability of *B. cinerea* to successfully infect its host [50,51,52]. Therefore, the observed defect in cell wall maintenance observed in *ΔBcMtp1* might be responsible for the weak virulence of *ΔBcMtp1* on various plant tissues. Further, the ability to adapt to changing osmotic conditions *in planta* was also demonstrated to be a critical process for *B. cinerea* virulence [44,53]. Accordingly, *ΔBcMtp1* exhibited increased sensitivity to osmotic stress, which may represent an additional reason why this mutant was compromised in virulence. The sensitivity of *ΔBcMtp1* to oxidative stress was also significantly increased, relative to the wild-type and complement strain controls. Reactive oxygen species (ROS) are a critical component of the plant-pathogen interaction. Plants potentiate oxidative bursts to rapidly enhance their defense against pathogens. Successful necrotrophs like *B. cinerea* rely upon a robust resistance to the antimicrobial effects of ROS to maintain virulence. In *B. cinerea*, this process is mediated through a series of oxidative stress detoxification systems including peroxidases and catalases [54,55]. Therefore, the increased sensitivity of *ΔBcMtp1* to oxidative stress might partially explain the weaker virulence of this mutant on host plants.

This reverse genetics approach to characterization clearly implicated BcMtp1 in various biological processes critical for normal function in *B. cinerea.* While vegetative development, asexual reproduction, fungicide sensitivity, pathogenicity, and stress tolerance were the phenotypic metrics we used to characterize this gene, we do not exclude the possibility that further phenotypes remain to be elucidated. A clear phenotypic characterization, however, is the first step towards identifying an exact biochemical mechanism. Without a clear understanding of the organism-wide effects of gene deletion, one cannot begin to logically infer which biochemical assays to perform, in the pursuit of that goal. Given the critical function that the BcMtp1 protein plays in almost every relevant phenotypic process, it does not escape our attention that this gene might represent an important target for future fungicides to be used in the control of *B. cinerea*.

## Figures and Tables

**Table 1:** Sensitivity of the wild-type progenitor B05.10 and the *BcMtp1* deletion mutants *ΔBcMtp1* and the complemented strain *ΔBcMtp1C* to different fungicides.

Strain	EC_50_ (μg mL^−1^) ^a^			
	Fluazinam	Boscalid	Fludioxonil	Tebuconazole
B05.10	0.02379 B	3.0572 A	0.0172 A	0.3985 A
*ΔBcMtp1*	0.2176 A	2.9714 A	0.0169 A	0.4057 A
*ΔBcMtp1C*	0.02209 A	3.0136 A	0.0162 A	0.3986 A

^a^ Data in the columns are the averages of three experiments. Values in the columns followed by the same letter are not significantly different according to a Fisher’s least significant difference (LSD) test at *p* = 0.05. Fluzinam (0, 0.0125, 0.025, 0.05, 0.1, or 0.2 μg ml^−1^), Fludioxonil (0, 0.00625, 0.0125, 0.025, 0.05, or 0.1 μg ml^−1^), Tebuconazole (0, 0.05, 0.1, 0.2, 0.4, or 0.8 μg ml^−1^), and Boscalid (0, 0.25, 0.5, 1, 2, or 4 μg ml^−1^).

## Data Availability

Not applicable.

## Supplementary Materials

The following supporting information can be downloaded at: https://www.mdpi.com/article/10.3390/jof9010025/s1, Figure S1: Alignment of BcMtp1 from Botrytis cinerea with those from Saccharomyces cerevisiae and other fungi. (A) The alignment of the amino acid sequences of BcMtp1 with those from *Aspergillus nidulans*, *Neurospora crassa*, *Candida albicans*, *Fusarium graminearum*, *Fusarium oxysporum*, *Magnaporthe oryzae*, *Sclerotinia sclerotiorum*, *Schizosaccharomyces pombe* and *Saccharomyces cerevisiae*. (B) Phylogenetic tree generated by the neighbour-joining method with Mega 4.1 software on the basis of the deduced amino acid sequences of BcMtp1 from Botrytis cinerea strain B05.10 and those from other fungi. Figure S2: Generation and identification of the BcMtp1 deletion mutant of *Botrytis cinerea*. (A) Gene replacement strategy for BcMtp1. The hygromycin resistance cassette (hph) is denoted by the large grey arrow. Primer (P3-P18) binding sites are indicated by arrows (see Table S1 for the primer sequences). (B) Southern blot hybridization analysis of strains using the 5′-flanking region of *BcMtp1* as a probe. Genomic DNA of the wild-type progenitor B05.10, BcMtp1 deletion mutant *ΔBcMtp1* and complemented strain *ΔBcMtp1C* were digested with *DraI*. Figure S3: Identification of the *BcMtp1* deletion mutant of *Botrytis cinerea* with PCR method. (A) Primer pair P11/P12 was used to specifically amplify the partial BcMtp1. (B) Primer pair P13/P14 was used to validate the se-lectable marker hph, Primer pairs P15/P16 and P17/P18 were used to amplify the two homologous arms with a partial fragment of the connecting area. Table S1: Primers used in this study.
