# Supplementary material for: Function of the Mitochondrial Transport Protein BcMtp1 in Regulating Vegetative Development, Asexual Reproduction, Stress Response, Fungicide Sensitivity, and Virulence of Botrytis cinerea
Source: J Fungi (Basel). 2022 Dec 23;9(1):25. doi: 10.3390/jof9010025 (PMC9864816; doi:10.3390/jof9010025)
Supplement: Supplementary file 1 [file jof-09-00025-s001.zip › jof-2096801-supplementary/JOF-supplemental photo and table -revised/Table S1_BcMtp1.docx]

**Table S1**

| Primer code Sequence (5′→3′) | Relevant characteristic |
| --- | --- |
| P1 ATGTCGGCCTCCAAGAATGAT | Amplify the full sequence of cDNA and genomic DNA the *BcMtp1* gene |
| P2 TCACAAGCCGGCATCTTCG |  |
| P3 ATTTGGGAGAGATGAGAGTGT | Amplify the left homologous arm of the *BcMtp1* gene of *B. cinerea* (1450 bp) |
| P4CCACCAGCCAGCCAACAGCTCCCTGGTAGGATATATGAGAACGT |  |
| P5 CAATACGCAAACCGCCTCTCCCCTATGGGAGCGGGCGTTATTTT | Amplify the right homologous arm of the *BcMtp1* gene of *B. cinerea* (1202 bp) |
| P6 GTACACGCCGAACGAACCACC |  |
| P7 GGGAGCTGTTGGCTGGCTGGTGG | Amplify the *hph* gene (1764 bp) |
| P8 GGGGAGAGGCGGTTTGCGTATTG |  |
| P9 TGGAGAAGAACCCAACACAT | Amplify the knockout vector of the  *BcMtp1* gene of *B. cinerea* (4203 bp) |
| P10 CAAAAGGCACTCAGAAGACG |  |
| P 11 TAAGAGCCCAAGGAGAT | Amplify a partial fragment of the *BcMtp1* gene of *B. cinerea* (459 bp) |
| P 12 AGCCAACCTTATCTGTTCG |  |
| P 13 actctattcctttgccctcgg | Amplify a partial fragment of the *hph* gene (475 bp) |
| P 14 GAAAAGTTCGACAGCGTCTCC |  |
| P 15 AGTCGTGAATAATCCAGCAA | Confirm whether the *hph* genes homologously replaced the *BcMtp1* gene of *B. cinerea* (2677bp) |
| P 16 GGAATCGGTCAATACACTA |  |
| P 17 gcaaagtgccgataaaca | Confirm whether the *hph* genes homologously replaced the  *BcMtp1* gene of *B. cinerea* (2524bp) |
| P 18 CACGACACGAGCACTA |  |
| P19 CGGGATCCCGCTTATGATGACTG | Amplify the *BcMtp1* gene (include the control region of the *BcMtp1* gene) (2149 bp) |
| P20 CCCAAGCTTAGCAGTATGATGAAAGGTGTG |  |
| P21 TTTTGGACAGGCGTATTA | Amplify a probe for Southern blotting (674 bp) |
| P22 ATAGAGCGAGCAACATCA |  |
| P25 TAGGTGATTTGGGACAACAGAG | Amplify the *bcyap1* gene for quantitative real-time PCR |
| P26 GTCTCTCAATGGTGCGGATAG |  |
| P27 GCTACGCCTATGGGAAGTAATG | Amplify the *bcmkk1* gene for quantitative real-time PCR |
| P28 CTACTTTCGCTTCCTCCACTTG |  |
| P29 GGCAAGTTGAAGGAGCAAATC | Amplify the *bcgls* gene for quantitative real-time PCR |
| P30 ATCTGGTGCGTGTGTGATAG |  |
| P31 GAAGGATGGAAGGGTCTTGTAG | Amplify the *BcERG11* gene for quantitative real-time PCR |
| P32 CTAACTCGGAGAAAGCCTGATG |  |
| P37 CGTCTGGATTGGTGGTTCTATT | Amplify the reference gene actin for quantitative real-time PCR |
| P38 ACTCGTCGTACTCTTGCTTTG |  |
