# Supplementary figures and images for: Function of the Mitochondrial Transport Protein BcMtp1 in Regulating Vegetative Development, Asexual Reproduction, Stress Response, Fungicide Sensitivity, and Virulence of Botrytis cinerea
Source: J Fungi (Basel). 2022 Dec 23;9(1):25. doi: 10.3390/jof9010025 (PMC9864816; doi:10.3390/jof9010025)

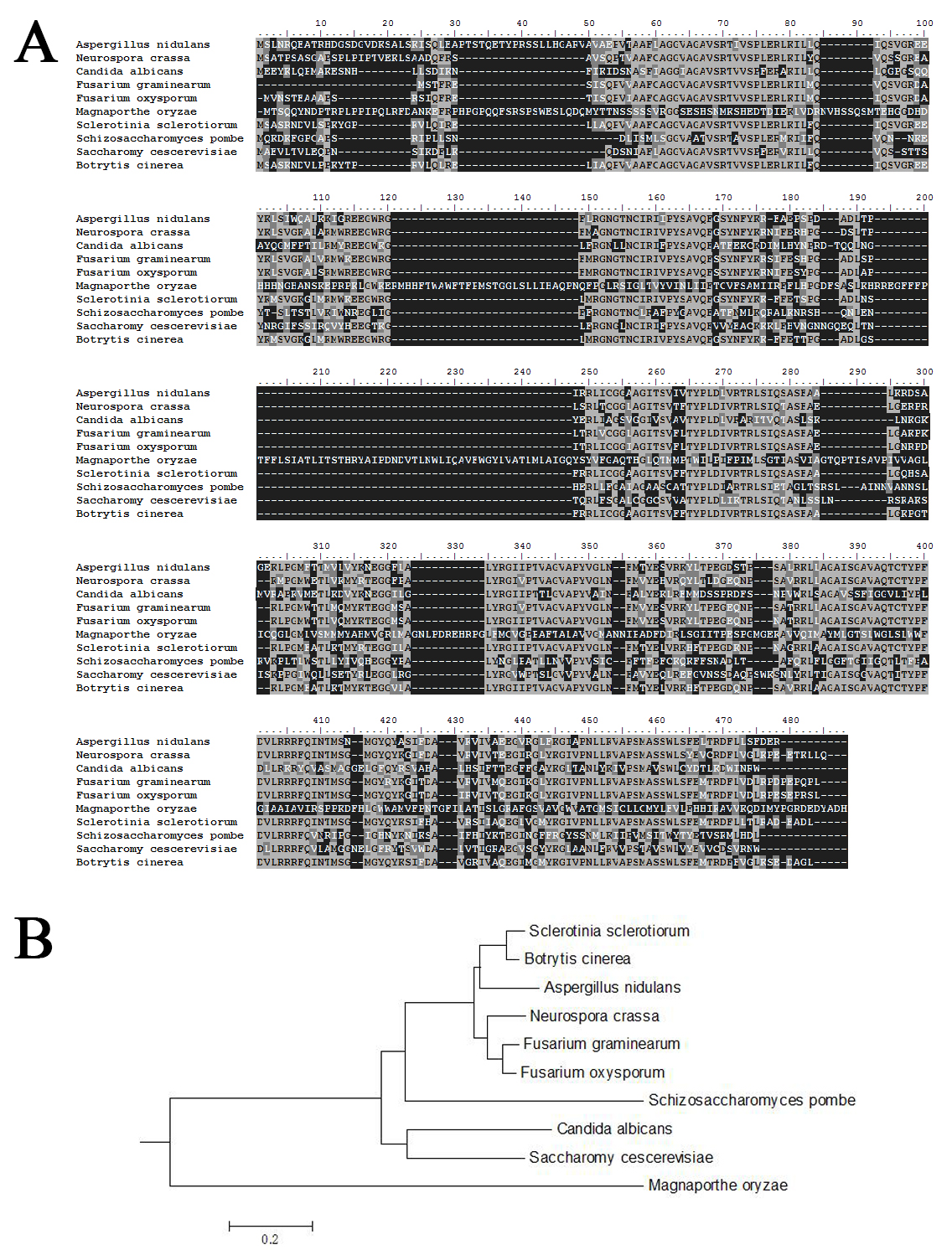

Supplement: Supplementary file 1 [file jof-09-00025-s001.zip › jof-2096801-supplementary/JOF-supplemental photo and table -revised/Figure S1-jof-revised.jpg]

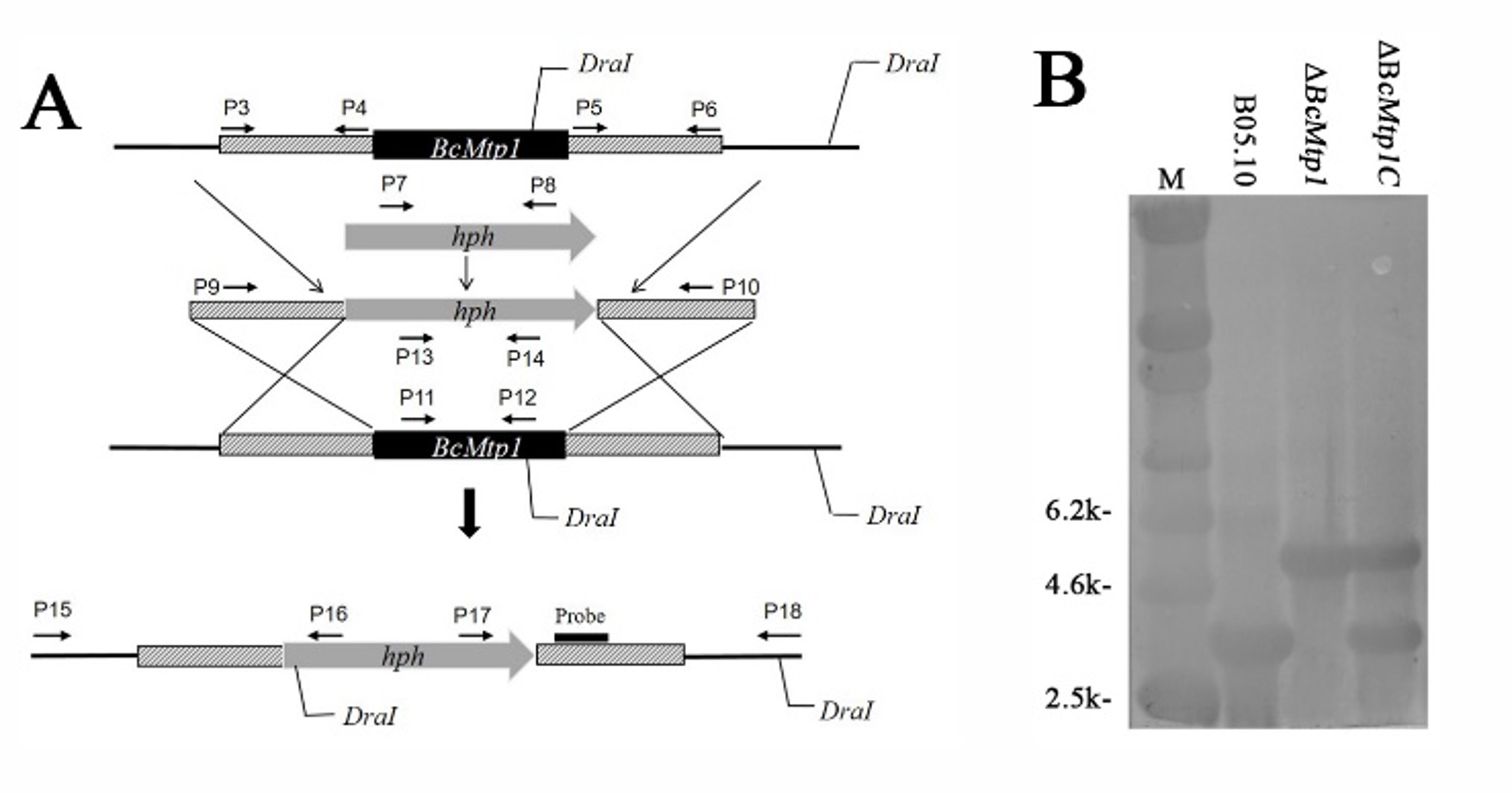

Supplement: Supplementary file 1 [file jof-09-00025-s001.zip › jof-2096801-supplementary/JOF-supplemental photo and table -revised/Figure S2-jof-revised.jpg]

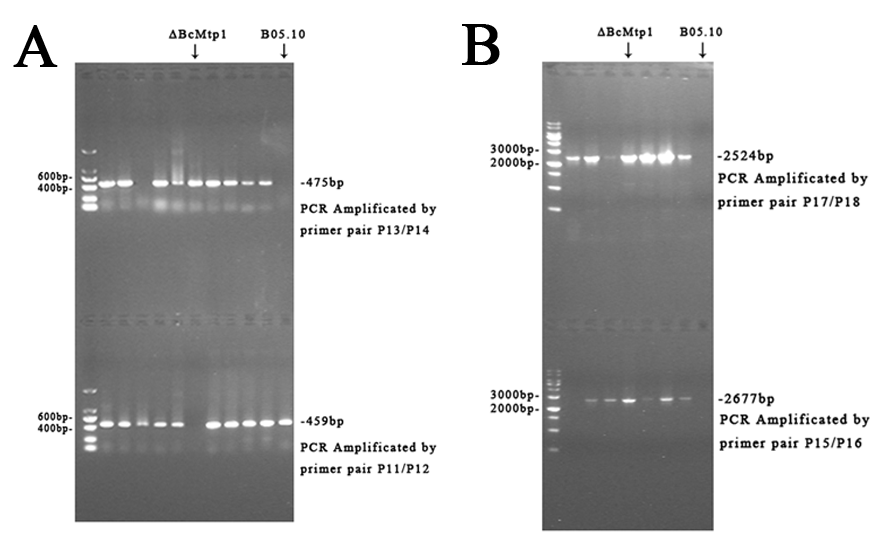

Supplement: Supplementary file 1 [file jof-09-00025-s001.zip › jof-2096801-supplementary/JOF-supplemental photo and table -revised/Figure S3-jof-revised.tif]
